# Dietary Vitamin A Improved the Flesh Quality of Grass Carp (*Ctenopharyngodon idella*) in Relation to the Enhanced Antioxidant Capacity through Nrf2/Keap 1a Signaling Pathway

**DOI:** 10.3390/antiox11010148

**Published:** 2022-01-12

**Authors:** Pei Wu, Li Zhang, Weidan Jiang, Yang Liu, Jun Jiang, Shengyao Kuang, Shuwei Li, Ling Tang, Wuneng Tang, Xiaoqiu Zhou, Lin Feng

**Affiliations:** 1 Animal Nutrition Institute, Sichuan Agricultural University, Chengdu 611130, China; wupei0911@sicau.edu.cn (P.W.); lilizhang09@gmail.com (L.Z.); WDJiang@sicau.edu.cn (W.J.); 11081@sicau.edu.cn (Y.L.); 2 Fish Nutrition and Safety Production University Key Laboratory of Sichuan Province, Sichuan Agricultural University, Chengdu 611130, China; 3 Key Laboratory for Animal Disease-Resistance Nutrition of China Ministry of Education, Sichuan Agricultural University, Chengdu 611130, China; 4 College of Animal Science and Technology, Sichuan Agricultural University, Chengdu 611130, China; jjun@sicau.edu.cn; 5 Animal Nutrition Institute, Sichuan Academy of Animal Science, Chengdu 610066, China; ksy_cd@163.com (S.K.); lishuwei84511614@126.com (S.L.); tingling@vip.163.com (L.T.); WNTANG@163.com (W.T.)

**Keywords:** vitamin A, flesh quality, grass carp, antioxidant, Nrf2 signaling

## Abstract

Fish is an important animal-source food for humans. However, the oxidative stress-induced by intensive aquaculture usually causes deterioration of fish meat quality. The nutritional way has been considered to be a useful method for improving fish flesh quality. This study using the same growth experiment as our previous study was conducted to investigate whether vitamin A could improve flesh quality by enhancing antioxidative ability via Nrf2/Keap1 signaling in fish muscle. Six diets with different levels of vitamin A were fed to grass carp (*Ctenopharyngodon idella*) (262.02 ± 0.45 g) for 10 weeks. Dietary vitamin A significantly improved flesh sensory appeal and nutritional value, as evident by higher pH_24h_ value, water-holding capacity, shear force, contents of protein, lipid, four indispensable amino acids (lysine, methionine, threonine, and arginine) and total polyunsaturated fatty acid in the muscle. Furthermore, dietary vitamin A reduced oxidative damage, as evident by decreased levels of muscle reactive oxygen species, malondialdehyde, and protein carbonyl, enhanced activities of antioxidative enzyme (catalase, copper/zinc superoxide dismutase (CuZnSOD), MnSOD, glutathione peroxidase, and glutathione reductase), as well as increased content of glutathione, which was probably in relation to the activation of nuclear factor erythroid 2-related factor 2 (Nrf2) signaling. These findings demonstrated that dietary vitamin A improved flesh quality probably by enhancing antioxidant ability through Nrf2/Keap 1a signaling in fish.

## 1. Introduction

Vitamin A, which is an unsaturated monohydric alcohol with β-ionone ring, is an essential nutrient for fish and capable of scavenging peroxyl radicals thus inhibiting lipid peroxidation in vitro [1]. Our previous study showed that vitamin A reduced the oxidative damage of lipid and protein in grass carp intestine [2]. To our knowledge, fish is an important animal-source food providing essential nutrients with high bioavailability, such as balanced amino acid and omega 3 long-chain polyunsaturated fatty acids (n-3 LC PUFA) for humans [3]. However, fish meat quality could be deteriorated by oxidative stress that is usually caused by intensive aquaculture. The nutritional way has been considered to be a useful method for enhancing fish flesh quality. Whether vitamin A could enhance fish flesh quality via its antioxidative benefits is not known so far.

It is well known that sensory property is one of the main sets of characteristics that make up fish flesh quality as perceived by the consumer [4]. Sensory acceptability of meat is primarily determined by water-holding capacity (WHC) and tenderness, which can be affected by pH value [5]. However, muscle pH value and WHC were reduced by oxidative stress in common carp (*Cyprinus carpio*) [6]. To date, no report has shown the effect of vitamin A on sensory appeal of fish flesh in relation to antioxidant capacity. In terrestrial animals, dietary vitamin A improved the juiciness and tenderness of longissimus thoracis from Holstein bulls and steers [7], the tenderness of longissimus lumborum from Angus crossbred steers [8], and increased the shear force, and decreased the pH_24h_ and drip loss of breast muscle in broiler [9]. Accordingly, dietary vitamin A might change the sensory appeal of fish flesh via antioxidation, which warrants further investigate.

In addition, nutritional value is another main characteristic of fish flesh quality [4]. A safe food supply provides nutritional benefit while posing minimal risks to consumers’ health [10]. However, protein oxidative change induces loss of essential amino acids and decrease in digestibility, ultimately reducing the nutritional quality of muscle [11], while lipid oxidation results in loss of nutrient value, off-flavor development, and accumulation of toxic compounds, which may be detrimental to the health of consumers [12]. Yet, little is known about whether vitamin A regulated nutritional value of fish flesh via antioxidant capacity. Studies have found that certain levels of dietary vitamin A enhanced the body crude protein content of juvenile hybrid tilapia (*Oreochromis niloticus* × *O. aureus*) [13], and enhanced the perirenal fat in finishing pigs [14]. Furthermore, certain contents of dietary vitamin A up-regulated fatty acid synthase mRNA level and enzyme activity in the liver of orange spotted grouper (*Epinephelus coioides*) [15]. Thus, vitamin A might affect the nutritional value of fish flesh, which awaits further characterization.

The present study used the same animal trial as our previous study, which reported that vitamin A deficiency depressed the growth and intestinal immunity of on-growing grass carp [16]. The present study aimed to investigate the effects of vitamin A on the flesh sensory appeal, nutritional quality, antioxidative ability, and the possible mechanisms in grass carp, which may be useful for elucidating the mechanisms whereby dietary vitamin A influenced muscle quality in fish. Additionally, the dietary vitamin A requirements for on-growing grass carp based on the muscle antioxidative parameters were also evaluated.

## 2. Materials and Methods

### 2.1. Animals, Diets, and Experimental Design

Healthy grass carp were procured from a commercial farm (Sichuan, China) and acclimatized to experimental conditions for four weeks in cages (1.4 m × 1.4 m × 1.4 m). Following another two weeks of vitamin A depletion period, 540 similarly-sized fish (262.02 ± 0.45 g) were randomly allocated into eighteen cages (three cages per treatment) and fed with six experimental diets differing in vitamin A content (18.69 (un-supplemented control), 606.8, 1209, 1798, 2805, and 3796 IU/kg), respectively. The experimental diets were formulated by supplementing retinyl acetate (500,000 IU/g) at concentrations of 0 (un-supplemented control), 600, 1200, 1800, 2800, and 3800 IU/kg into the basal diet (Table 1), which contained 30% crude protein according to the study of Khan et al. [17]. The dietary vitamin A contents were assayed by high-performance liquid chromatographic (HPLC) as described by Moren et al. [18]. During the 70 days experimental period, the fish were kept in natural light and dark cycle, and fed four times daily to apparent satiation. Uneaten feed was removed by using a disc equipped in the bottom of cage. Water temperature was 28 ± 2 °C, pH 7.0 ± 0.2, and dissolved oxygen ≥ 6.0 mg/L.

### 2.2. Sample Collection and Biochemical Analysis

Fishes were anaesthetized with benzocaine before sampling, following procedures from Chen et al. [20]. After sacrifice, the left-side muscle of two fish in each cage were quickly manually filleted, frozen, and preserved at −80 °C until needed for analysis, while the right-side muscle was used for analysis of sensory appeal parameters. Parts of the muscle were fixed in 10% neutral formalin for morphological observation, similar to the previous study from our laboratory [2]. pH value, cooking loss, and shear force of fish muscle were assayed according to Brinker and Reiter [21]. Briefly, muscle pH value was detected using a calibrated pH probe (Testo AG Company, Lenzkirch, Germany) after slaughter and then at 24 h post-mortem (pH_24h_). Cooking loss was determined by weight changes before and after cooking (sealed in PE-bag and heated at 70 °C for 20 min). Flesh shear force was assayed using an Instron 4411 material testing instrument (Instron Corporation, Canton, MI, USA). Proximate composition, free amino acids, and fatty acids contents of muscle were analyzed according to the method of AOAC [22], using a L-8800 amino acid analyzer (Hitachi Ltd., Tokyo, Japan) and gas chromatography method similar to Carbonera et al. [23], respectively.

For antioxidant-related parameters assay, muscle tissue homogenates were prepared according to the kit instructions. Reactive oxygen species (ROS), malondialdehyde (MDA), protein carbonyl (PC), anti-superoxide anion (ASA) and anti-hydroxyl radical (AHR), superoxide dismutase (SOD), catalase (CAT), glutathione peroxidase (GPx), glutathione-S-transferase (GST), and glutathione reductase (GR), as well as reduced glutathione (GSH) were determined using the commercial detection kits (Nanjing Jiancheng Bioengineering Institute, Nanjing, China). Activities of cathepsin B and L were assayed by the method described by Bahuaud et al. [24]. Measurement of hydroxyproline, lactic acid, and carnosine contents in muscle followed Periago et al. [25], Hultmann et al. [26], and Elbarbary et al. [27], respectively. Muscle vitamin A contents were measured by HPLC method.

### 2.3. Histology Observation

Histological observation of muscle was performed as described in our previous study [28]. In brief, the fixed muscle tissues were dehydrated in alcohol and embedded in paraffin wax. Afterwards, the tissues were serially sectioned to 4 mm, and stained with hematoxylin and eosin (H & E). The muscle morphological was observed using a Nikon TS100 light microscope (Nikon, Tokyo, Japan).

### 2.4. Quantitative Real-Time PCR Analysis

Procedures of total RNA isolation, reverse transcription, and quantitative real-time PCR (qPCR) were similar to our previous study [16]. In brief, an RNAiso Plus kit (Takara, Dalian, China) was used to isolate total RNA from muscle. One percent agarose gel electrophoresis and spectrophotometric analysis (A260: 280 nm) were used to assay RNA purity and concentration, respectively. After this, cDNA synthesis was performed using a PrimeScript^TM^ RT reagent Kit (Takara, Dalian, China). Finally, qPCR was performed using a CFX96^TM^ Real-Time PCR System (Bio-Rad, Hercules, CA, USA) with SYBR Green (Takara, Dalian, China). According to the preliminary experiment, β-actin was chosen as the reference gene (data not shown). Specific primers for qPCR were listed in Table 2. The amplification efficiencies of all primers were verified to be approximately 100%. The relative gene expressions were analyzed using the 2^−ΔΔCT^ method as described by Livak and Schmittgen [29].

### 2.5. Western Blotting Measurement

The procedure of Western blotting was the same as previous study from our laboratory [30]. Shortly thereafter, extracted total and nuclear protein concentrations from muscle were measured using a protein quantification kit (Bio-Rad, Hercules, CA, USA). After this, protein samples with equal amounts were separated by SDS-PAGE and transferred to a PVDF membrane. Membranes were blocked at room temperature for 1 h, incubated with primary antibody at 4 °C overnight, and then with HRP-conjugated secondary antibodies for 2 h. Anti-Nrf2, total TOR (T-TOR), phospho-TOR Ser2448 (p-TOR), β-actin, and lamin B1 antibodies were the same as previous studies from our laboratory [31,32]. Finally, the bands were visualized and quantified by using an ECL kit (Millipore, Billerica, MA, USA) and Image J software (NIH, Bethesda, MD, USA), respectively. Protein levels in vitamin A supplemented groups were expressed relative to those in the vitamin A-deficient group. The analysis was repeated three times, and similar results were obtained each time.

### 2.6. Statistical Analysis

Data were treated by using Excel 2019 (Microsoft Inc., Redmond, WA, USA). The data from the individual fish in the same replicate were averaged, and then this mean for the replicate was used in the analysis. Prior to any statistical analysis, normality and homoscedasticity assumptions were confirmed. One-way ANOVA followed by Tukey’s HSD test was used for vitamin A effects statistical analyses with SAS 9.4 (SAS Institute Inc., Cary, NC, USA). *p*-value < 0.05 was considered as statistically significant. The linear and quadratic effect of vitamin A were assayed by orthogonal polynomial contrasts in SAS 9.4. The results are presented as mean and SEM. Data visualization was performed by using the GraphPad Prim 8.0 (GraphPad Inc., La Jolla, CA, USA) and Excel 2019 (Microsoft Inc., Redmond, WA, USA).

## 3. Results

### 3.1. Proximate Compositions and Physicochemical Characteristics of Muscle

As presented in Table 3, contents of crude protein and crude lipid in muscle were linearly (*p* < 0.05) and quadratically (*p* < 0.05) enhanced by increase in dietary VA, and the highest in the group with 1798 IU/kg VA, while muscle moisture content was linearly (*p* < 0.05) and quadratically (*p* < 0.05) reduced with increase in dietary VA contents, and the lowest in the group with 1798 IU/kg VA. Compared to the VA deficiency group, increased levels of dietary VA linearly (*p* < 0.05) and quadratically (*p* < 0.05) enhanced shear force, pH_24h_, as well as carnosine content in grass carp muscle, while linearly (*p* < 0.05) and quadratically (*p* < 0.05) reduced cooking loss, lactic acid content, and cathepsin B and L activities in muscle. Meanwhile, muscle hydroxyproline content showed a quadratically (*p* < 0.05) increase as dietary VA levels increased, and were the highest in the groups with 1209 and 1798 IU/kg VA.

### 3.2. Free Amino Acid Contents and Fatty Acid Profile in Muscle

In order to determine the effects of VA on flesh flavor, we focused on the free amino acid contents in grass carp muscle. The results showed that the free lysine, methionine, glutamic acid, threonine, and arginine contents in muscle were linearly (*p* < 0.05) and quadratically (*p* < 0.05) increased as dietary VA levels increased. As for lysine and arginine contents, they were significantly increased with increase in dietary VA levels up to 1209 IU/kg (*p* < 0.05), and then plateaued. Methionine content in group with 1798 IU/kg VA was significantly higher than that in the VA deficiency group (*p* < 0.05). Threonine content was significantly improved with the increasing VA levels up to 606.8 IU/kg (*p* < 0.05), and plateaued thereafter. Glutamic acid content was significantly enhanced by 606.8–3796 IU/kg VA (*p* < 0.05), and the highest in the group with 1798 IU/kg VA. However, the other amino acids contents were not significantly affected by dietary VA (Table 4).

Fatty acids are important precursors of flesh flavor. Accordingly, we also evaluated the muscle fatty acids profile. We observed that dietary VA linearly (*p* < 0.05) enhanced the C18: 3n − 3, C20: 3n − 3, C22: 6 (docosahexaenoic acid, DHA) and total polyunsaturated fatty acid (PUFA) contents in muscle, linearly (*p* < 0.05) and quadratically (*p* < 0.05) enhanced the total unsaturated fatty acid content in muscle, whereas linearly (*p* < 0.05) reduced muscle C160 content, and linearly (*p* < 0.05) and quadratically (*p* < 0.05) decreased the C16:1 and total saturated fatty acid contents in muscle (Table 5). C16:1 content significantly decreased with increase in dietary VA levels up to 1209 IU/kg, and plateaued thereafter. Compared to the VA deficiency group, C18: 3n − 3 was significantly higher in groups with 2805 and 3796 IU/kg VA (*p* < 0.05), and C20: 3n − 3, C22: 6, ΣUFA and ΣPUFA contents were significantly higher in group with 2805 and 1798 IU/kg VA (*p* < 0.05), respectively, while ΣSFA content was significantly lower in group with 1798 IU/kg VA (*p* < 0.05).

### 3.3. Antioxidant Related Parameters in Muscle

**Figure 1:**
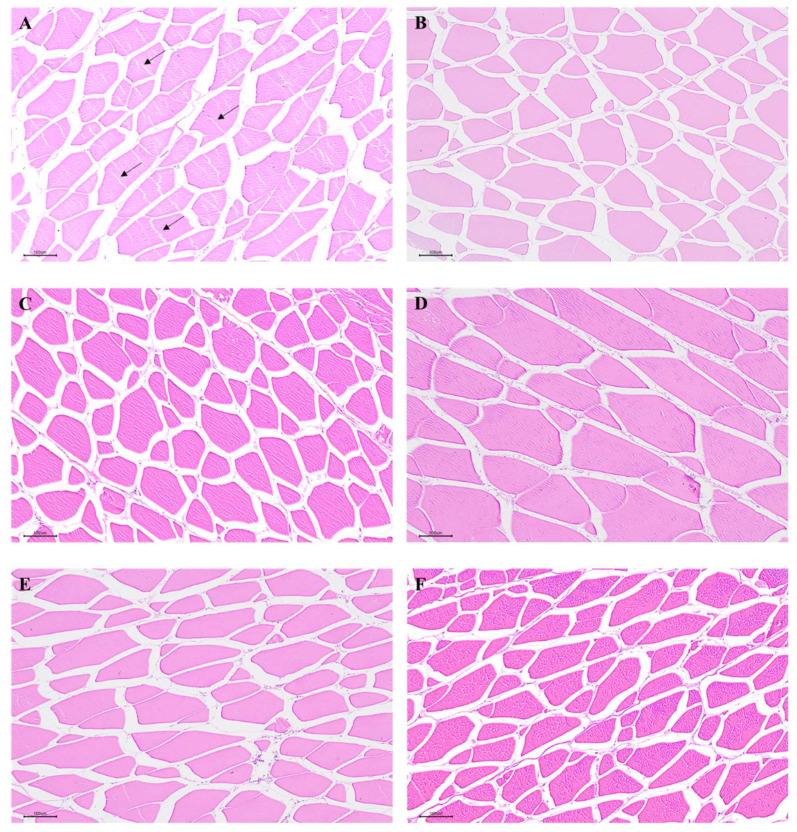
The histology of on-growing grass carp muscle (H&E 100×): (**A**) The vitamin A-deficient group. Arrowhead showed the rupture in muscle fiber. (**B**) The group with vitamin A at 606.8 IU/kg. (**C**) The group with vitamin A at 1209 IU/kg. (**D**) The group with vitamin A at 1798 IU/kg. (**E**) The group with vitamin A at 2805 IU/kg. (**F**) The group with vitamin A at 3796 IU/kg.

**Figure 2:**
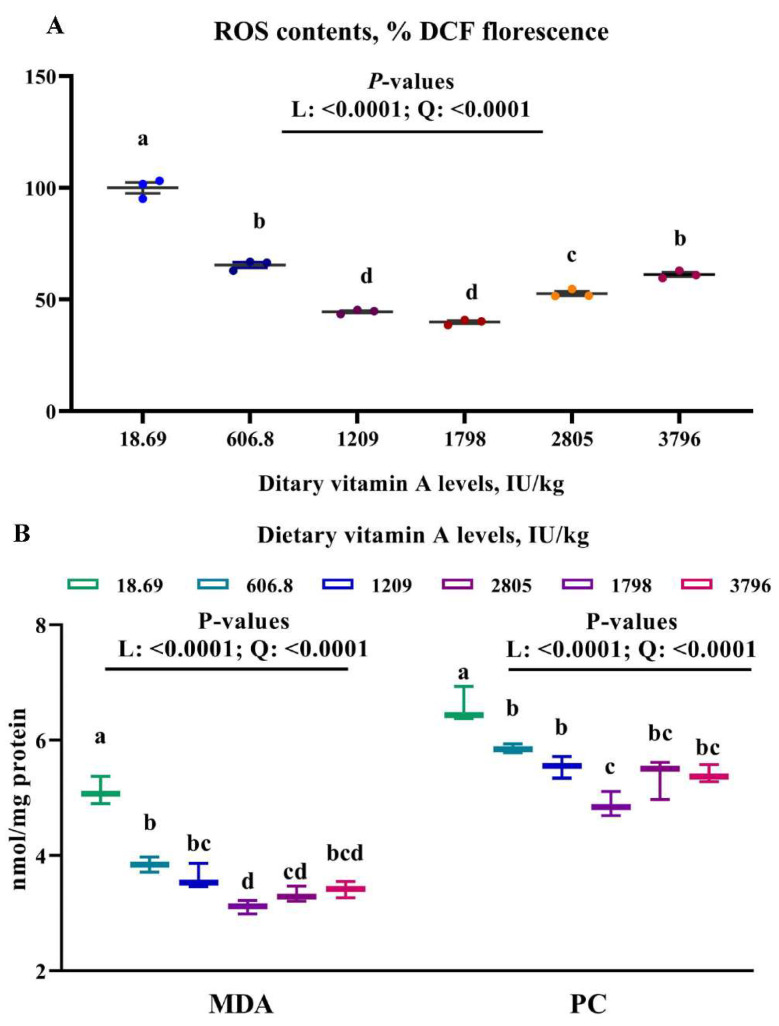
Effects of dietary vitamin A on contents of ROS (**A**), MDA and PC (**B**) in muscle of on-growing grass carp. Data are means ± SEM of three replicate groups, two fish for each replicate (*n* = 3). ^a,b,c,d^ within a column, means without a common lowercase superscript differ (*p* < 0.05). *p*-values underlined with a solid line indicate a linear and quadratic response to dietary vitamin A levels. SEM = standard error of the mean; L = linear; Q = quadratic; ROS = reactive oxygen species, %DCF florescence; MDA = malondialdehyde, nmol/mg prot; PC = protein carbonyl, nmol/mg prot.

To test whether dietary VA affected muscle antioxidant capacity, we analyzed antioxidant related parameters in muscle. The histological results indicated that an obvious rupture in the muscle fiber occurred in the dietary VA deficiency group, but was not observed in other groups (Figure 1). Furthermore, muscle MDA, PC, and ROS contents were linearly (*p* < 0.05) and quadratically (*p* < 0.05) reduced by the enhanced levels of dietary VA (Figure 2). Muscle CAT, CuZnSOD, GPx and GR activities, as well as GSH and VA content were linearly (*p* < 0.05) and quadratically (*p* < 0.05) improved by the increase in dietary VA levels, while ASA, AHR capacities, and MnSOD activity showed a quadratically (*p* < 0.05) enhancement as dietary VA increased (Table 6). However, GST activity in muscle was not significantly affected by dietary VA. Muscle ROS, MDA, and PC contents were significantly reduced by dietary VA supplementation in comparison with the VA deficiency group (*p* < 0.05), and was the lowest in the group with 1798 IU/kg VA. Compared to the VA deficiency group, supplementation of 1209 and 1798 IU/kg VA significantly enhanced the ASA and AHR capacities in muscle (*p* < 0.05). MnSOD and GR activities in group with 1798 IU/kg VA (*p* < 0.05) was significantly higher than those in the VA deficiency group (*p* < 0.05). CuZnSOD, CAT, and GPx activities, and GSH content in muscle, significantly increased with the increase in dietary VA levels up to 1209 IU/kg (*p* < 0.05), and plateaued thereafter. Muscle VA content was the highest in the group with 3796 IU/kg VA (*p* < 0.05).

**Figure 3:**
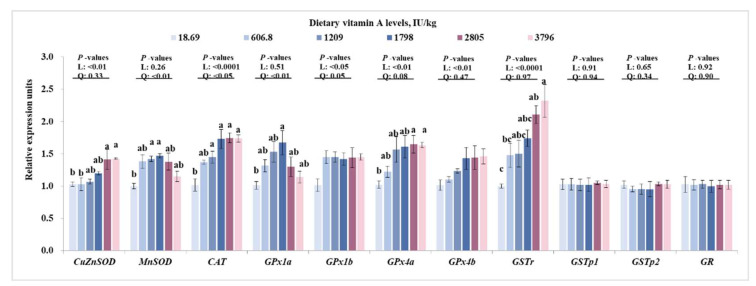
Effects of dietary vitamin A on relative mRNA levels of antioxidant enzymes genes in muscle of on-growing grass carp. Data are means ± SEM of three replicate groups, two fish for each replicate (*n* = 3). ^a, b, c^ within a column, means without a common lowercase superscript differ (*p* < 0.05). *p*-values underlined with a solid line indicate a linear and quadratic response to dietary vitamin A levels. SEM = standard error of the mean; L = linear; Q = quadratic; CuZnSOD = copper/zinc superoxide dismutase; MnSOD = manganese superoxide dismutase; CAT = catalase; GPx1a = glutathione peroxidase 1a; GPx1b = glutathione peroxidase 1b; GPx4a = glutathione peroxidase 4a; GPx4b = glutathione peroxidase 4b; GSTr = glutathione-S-transferase r; GSTp1 = glutathione-S-transferase p1; GSTp2 = glutathione-S-transferase p2; GR = glutathione reductase.

To fully characterize the effects of dietary VA on antioxidative enzymes, we tested the relative expressions of antioxidant enzymes genes in muscle. As presented in Figure 3, dietary VA linearly (*p* < 0.05) and quadratically (*p* < 0.05) up-regulated the relative gene expressions of *CAT* and *GPx1b* in muscle, linearly (*p* < 0.05) improved the relative mRNA levels of *CuZnSOD*, *GPx4a, GPx4b,* and *GSTr* in muscle, and quadratically (*p* < 0.05) increased the relative gene expressions of *MnSOD* and *GPx1a*, but did not significantly affect the relative mRNA levels of *GSTp1*, *GSTp2* and *GR*. Compared to the VA deficiency group, the relative mRNA levels of *CuZnSOD, GPx4a,* and *GSTr* were significantly higher in groups with 2805 and 3796 IU/kg VA (*p* < 0.05), the relative mRNA levels of *MnSOD* and *GPx1a* were significantly higher in groups with 1209 and/or 1798 IU/kg VA (*p* < 0.05). The relative mRNA levels of *CAT* in muscle significantly increased with increase in dietary VA levels up to 1209 IU/kg (*p* < 0.05), and then plateaued.

### 3.4. Nrf2 and TOR Signaling in Muscle

**Figure 4:**
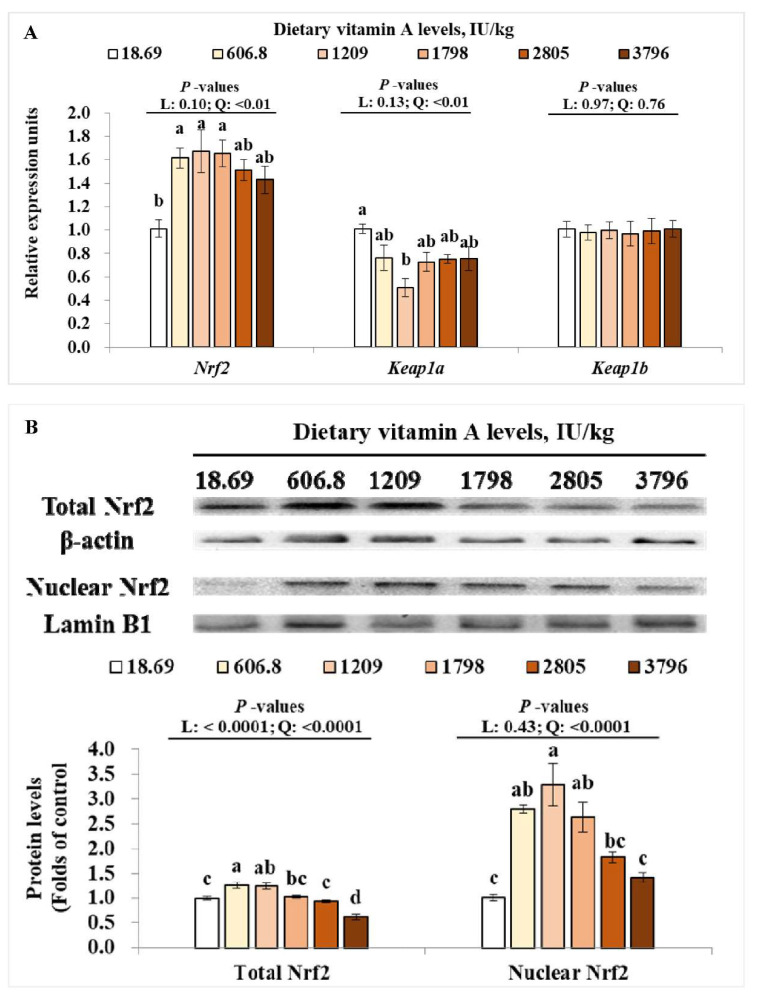
Effects of dietary vitamin A on relative mRNA levels of *Nrf2*, *keap1a,* and *keap1b* (**A**); total and nuclear levels of Nrf2 protein (**B**) in muscle of on-growing grass carp. Data are means ± SEM of three replicate groups, two fish for each replicate (*n* = 3). ^a,b,c,d^ within a column, means without a common lowercase superscript differ (*p* < 0.05). *p*-values underlined with a solid line indicate a linear and quadratic response to dietary vitamin A levels. SEM = standard error of the mean; L = linear; Q = quadratic; Nrf2 = nuclear factor erythroid 2-related factor 2; Keap1a = Kelch-like ECH-associated protein 1a; Keap1b = Kelch-like ECH-associated protein 1b.

To clarify the signaling involved in VA-regulated antioxidant capacity, we studied the TOR and Nrf2 signaling in muscle. For Nrf2 signaling pathway, as dietary VA increased, *Nrf2* gene expressions in the muscle were quadratically (*p* < 0.05) increased, while Kelch-like ECH-associating protein a (*Keap1a*) gene expression was quadratically (*p* < 0.05) down-regulated (Figure 4A). However, the relative mRNA levels of *Keap1b* in muscle were not significantly changed by dietary VA (Figure 4A). Furthermore, the total Nrf2 protein level in muscle was linearly (*p* < 0.05) and quadratically (*p* < 0.05) increased by increasing levels of dietary VA, and the nuclear Nrf2 level in muscle was quadratically (*p* < 0.05) enhanced by increase in dietary VA (Figure 4B). Compared to the VA deficiency group, the relative mRNA levels of *Nrf2*, protein levels of nuclear Nrf2 and total Nrf2 were significantly higher in groups with 606.8, 1209, and/or 1798 IU/kg VA (*p* < 0.05), while the relative mRNA levels of *Keap1a* was significantly lower in group with 1209 IU/kg VA (*p* < 0.05).

**Figure 5:**
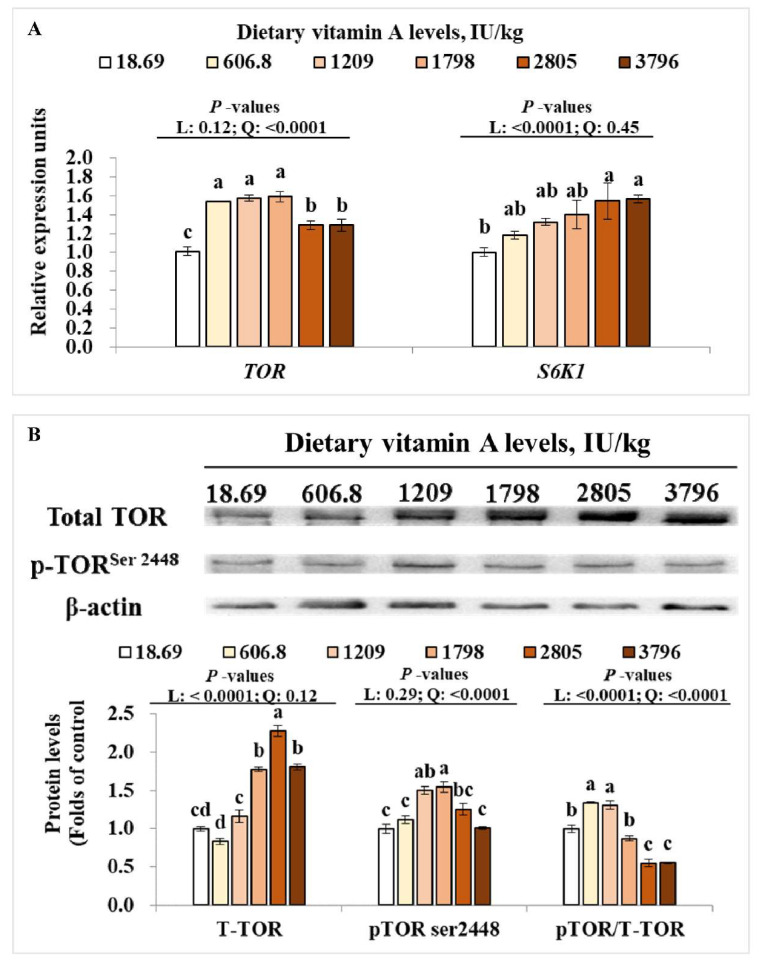
Effects of dietary vitamin A on relative mRNA levels of *TOR* and *S6K1* (**A**), total and phosphorylation levels of TOR protein (**B**) in muscle of on-growing grass carp. Data are means ± SEM of three replicate groups, two fish for each replicate (*n* = 3). ^a,b,c,d^ within a column, means without a common lowercase superscript differ (*p* < 0.05). *p*-values underlined with a solid line indicate a linear and quadratic response to dietary vitamin A levels. SEM = standard error of the mean; L = linear; Q = quadratic; TOR = target of rapamycin; S6K1 = ribosomal protein s6 kinase polypeptide 1.

As presented in Figure 5A, dietary vitamin A quadratically (*p* < 0.05) and linearly (*p* < 0.05) up-regulated the relative gene expressions of *TOR* and *S6K1* in muscle, respectively. Meanwhile, the protein levels of T-TOR and p-TOR^ser2448^ in muscle were linearly (*p* < 0.05) and quadratically (*p* < 0.05) enhanced by dietary VA, respectively. Muscle p-TOR/T-TOR was linearly (*p* < 0.05) and quadratically (*p* < 0.05) changed by dietary VA (Figure 5B). Compared to the VA deficiency group, the relative gene expressions of *TOR* and *S6K1* in muscle were significantly up-regulated by 606.8-3796 and 2805-3796 IU/kg VA (*p* < 0.05), respectively, the protein levels of T-TOR and p-TOR^ser2448^ were significantly higher in groups with 1798-3796 and 1209-1798 IU/kg VA (*p* < 0.05), respectively.

### 3.5. Dietary Vitamin A Requirements for On-Growing Grass Carp

**Figure 6:**
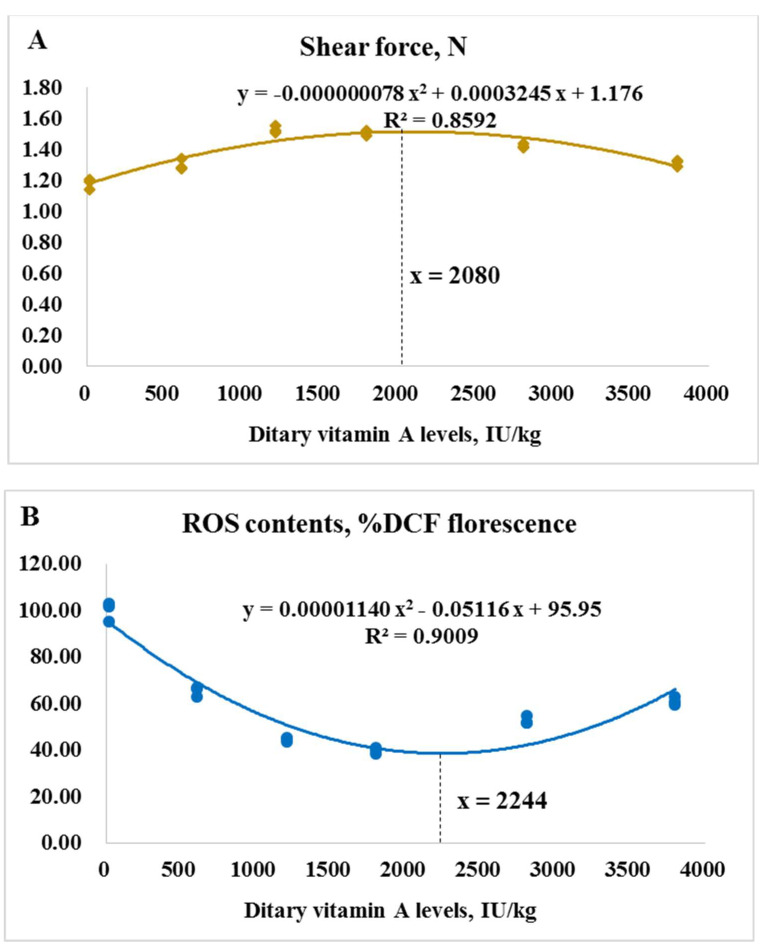
The dietary vitamin A requirements for on-growing grass carp based on muscle shear force (**A**) and contents of reactive oxygen species (ROS, (**B**)).

As presented in Figure 6, based on muscle shear force and ROS contents, the dietary VA requirements for on-growing grass carp (262.02–996.67 g) were determined to be 2080 and 2244 IU/kg diet, respectively.

## 4. Discussion

This present study used the same growth experiment as our previous research [2,16], which indicated that VA deficiency induced poor growth, impaired intestinal antioxidant capacity, induced apoptosis, and depressed intestinal immunity of on-growing grass carp. Oxidant damage usually can cause deterioration of fish flesh quality. Therefore, the present study focused on clarifying whether dietary VA could improve fish flesh quality through increasing antioxidative capacity.

### 4.1. Vitamin A Improved Fish Flesh Quality

One of the main sets of flesh quality characteristics is sensory appeal, which can be reflected by WHC, tenderness, and pH value [5]. In the present study, dietary VA deficiency induced a drop of shear force and pH_24h_ value, as well as an increase in cooking loss in grass carp muscle; however, optimal levels of dietary VA reversed these undesirable changes, indicating that VA is helpful for the improvement of fish flesh quality. Although no more information about the effects of VA on sensory appeal was found in fish, our results were similar to studies in terrestrial animals, which indicated that dietary VA improved juiciness and tenderness of longissimus thoracis from Holstein bulls and steers [7], tenderness of longissimus lumborum from Angus crossbred steers [8], and the sensory appeal of breast muscle in broiler [9]. In fish, the decrease in muscle pH value is closely related to the lactic acid production [33]. The present study found that lactic acid content in grass carp muscle was reduced by dietary VA. Meanwhile, correlation analysis showed that muscle pH_24h_ value was negative in relation to lactic acid content (Table 7), indicating that VA-increased muscle pH_24h_ value was partly ascribed to the lactic acid reduction in muscle. Muscle firmness is positively associated with collagen content (reflected by hydroxyproline content) [34], and negatively associated with activities of cathepsins B and L, which play important roles in post-mortem degradation of tissue proteins [35] and are positively correlated to detachments in the muscle structure in fish [26]. In this study, dietary VA enhanced the hydroxyproline content and decreased the cathepsin B and L activities in grass carp muscle. Further analysis indicated that muscle shear force was positively related to hydroxyproline content and negatively related to cathepsin B and L activities (Table 7), suggesting that VA increased fish muscle firmness partly through enhancing collagen content and decreasing cathepsin B and L activities.

The nutritional composition of muscle is another major quality aspect in fresh fish. Meanwhile, muscle free amino acids and fatty acids are major flavor contributors and important flavor precursors in fish, respectively [4]. Data in this study showed that contents of muscle protein, lipid, some free amino acids (Lys, Met, Thr, Arg, and Glu), DHA, and total PUFA were decreased by the VA deficiency and increased by optimal levels of dietary VA, suggesting that VA improved muscle nutritional composition of fish. These results were similar to studies that indicated that dietary VA increased body crude protein content of juvenile hybrid tilapia [13], and perirenal fat in finishing pigs [14]. However, information available on the effects of VA on amino acids and fatty acids contents in fish is scarce. Elongases of very long-chain fatty acid (Elovl) are involved in LC-PUFA biosynthesis by catalyzing the rate-limiting condensation step in elongation process [36]. Study found that VA upregulated fatty acid elongase-4 (Elovl4) mRNA levels in WNIN/Ob obese rat retina [37]. This might partially explain the VA-increased muscle DHA and PUFA contents in the present study.

### 4.2. Vitamin A Enhanced the Muscle Antioxidant Capacity of Fish

Lipid and protein oxidation, not only is one of the major causes of meat quality deterioration [12], but it also leads to damage of the structural integrity of cells [38]. High unsaturated lipid levels in fish flesh make it more susceptible to oxidative deterioration, which is mainly caused by an imbalance between ROS production and antioxidative defense [12]. MDA and PC are good biomarkers of protein oxidation and lipid peroxidation in animal tissues [39]. In the present study, dietary VA deficiency-induced rupture in the muscle fiber, while VA supplementation groups did not show this change. Furthermore, compared with the VA-deficient group, optimal levels of VA reduced ROS, MDA, and PC contents in grass carp muscle, showing that VA depressed the oxidative damage of fish muscle. Superoxide anion (O_2_^•−^) and hydroxyl radical (•OH−) are two important toxic ROS involving in oxidative damage [40]. Antioxidant enzymes (like SOD and GPx) and non-enzymatic antioxidant (like GSH) play important roles in free radicals scavenging. SOD is the main element of the first level of antioxidant defense against superoxide radical, CAT, and GPx play important roles in detoxifying the hydrogen peroxide, GST, and GR are also important glutathione-dependent enzymes and able to counteract the peroxidative damage [39]. Meanwhile, GSH is a low-molecular-mass thiol that involves in scavenging peroxyl radicals in cells [39]. In this study, VA deficiency decreased superoxide anion and hydroxyl radical scavenging capacities (ASA and AHR, respectively), CAT, CuZnSOD, MnSOD, GPx, and GR activities, as well as GSH and VA contents in grass carp muscle, while optimal levels of vitamin A reversed these changes, indicating that vitamin A decreased ROS content possibly via improving the antioxidant defense in fish muscle. However, GST activity in muscle was not significantly affected by dietary VA in this study. This result was different from the previous study from our laboratory, which found that VA deficiency reduced intestinal GST activity in grass carp [2]. The different results between muscle and intestine might be partly related to the tissue distribution of GST. In river pufferfish (*Takifugu obscurus*), the *GST* genes expressions in intestine were higher than those in muscle, which is associated with the fact that intestine is more susceptible to oxidant damage than muscle [41]. However, further investigation is necessary to clarify the exact mechanisms behind these findings.

As we know, activities of antioxidative enzymes were partly relied on their gene expression. In the current study, dietary VA deficiency resulted in a decrease in *CAT*, *CuZnSOD*, *MnSOD*, *GPx1a*, *GPx4a* and *GSTr* mRNA levels in muscle, which might partly explain the decrease in CAT, CuZnSOD, MnSOD, and GPx activities in the VA-deficient group. However, *GSTp1*, *GSTp2,* and *GR* mRNA levels in muscle showed no difference among groups. The difference change pattern of *GST* isoforms in muscle might be partly related to their tissue-specifically expression. In bighead carp (*Aristichthys nobilis*), the relative gene expression of *GST rho* was higher than that of *GST pi* in muscle [42]. The higher expression of *GSTr* in muscle might lead to the more sensitive response to VA; however, this needs further characterization. In mammal, the expression of antioxidative enzymes genes (such as *CAT* and *CuZnSOD*) can be modulated by Nrf2 signaling [43]. The present study observed that dietary VA deficiency decreased mRNA levels of *Nrf2*, protein levels of total Nrf2, and nuclear Nrf2, while enhancing the mRNA levels of *Keap1a* in grass carp muscle. These results demonstrated that VA deficiency-decreased antioxidative capacity might partly be attributed to the down-regulated Nrf2 levels. However, the muscle *Keap1b* mRNA level was not influenced by VA. This is similar with our previous studies, which found that VA deficiency upregulated *Keap 1a* gene expressions rather than *Keap 1b* in intestine, head kidney, and spleen of grass carp [2,44], demonstrating that VA regulated antioxidant gene expressions mainly via Nrf2/Keap 1a signaling in fish with unknown mechanisms. Additionally, in vitro study showed that inhibition of TOR reduced total Nrf2 expression in human hepatic carcinoma cells [45]. Our current results indicated that dietary VA enhanced the mRNA levels of *TOR* and *S6K1*, and total protein and phosphorylation levels of TOR in muscle of grass carp, which showed similar change patterns as total Nrf2 and nuclear Nrf2 levels, implying that VA-activated Nrf2 signaling might partly be due to the activation of TOR in muscle.

### 4.3. Vitamin A Requirement for On-Growing Grass Carp

As demonstrated by the above data, VA deficiency could lead to the deteriorated muscle quality of on-growing grass carp probably via depressing antioxidant ability in muscle. Accordingly, it is valuable to estimate the VA requirements for grass carp based on the muscle quality indices. Based on muscle shear force, contents of ROS, the VA requirements for on-growing grass carp (262.02–996.67 g) were determined to be 2080 and 2244 IU/kg diet, respectively, which were higher than that based on growth (1929 IU/kg diet), suggesting that more VA might be required for improving fish muscle quality. It is consistent with the study that showed that on-growing grass carp had higher folic acid requirements based on flesh quality [46]. Meanwhile, the VA requirements for on-growing grass carp based on healthy indices of intestine, head kidney, and spleen were higher than that based on growth [16,44]. These results demonstrated that higher dietary intake of nutrients is often necessary to satisfy physiology functions other than growth in fish.

## 5. Conclusions

The present study demonstrated that dietary VA depressed deterioration of sensory appeal, nutrition value, as well as flavor quality of fish flesh probably through improving antioxidant capacity, as shown by increased activities and gene expressions of SOD, CAT, and GPx, which was closely related to Nrf2/Keap 1a (rather than Keap 1b) signaling in muscle. However, VA showed different impacts on GST and *GST* isoforms in muscle with unknown mechanisms. In addition, the dietary VA requirements for improving flesh quality of on-growing grass carp were determined to be 2080–2244 IU/kg, which was slightly higher than that based on growth (1929 IU/kg).

## Figures and Tables

**Table 1:** Formulation and nutrient content of the basal diet.

Ingredients	%	Nutrients Content	%
Fish meal	15.55	Crude protein ^4^	29.71
Soybean protein concentrate	26.25	Crude lipid ^4^	3.58
Gelatin	3.13	n-3 ^5^	0.50
α-starch	24.00	n-6 ^5^	1.00
Maize starch	16.32	Available phosphorus ^5^	0.84
Soybean oil	1.93		
Cellulose	5.00		
L-Met (98%)	0.40		
Ca(H_2_PO_4_)_2_	2.87		
Vitamin premix ^1^	1.00		
Mineral premix ^2^	2.00		
Vitamin A premix ^3^	1.00		
Choline chloride (60%)	0.50		
Ethoxyquin (30%)	0.05		

^1^ Per kilogram of vitamin premix (g/kg): cholecalciferol (172 mg/g), 0.40; DL-α-tocopherol acetate (50%), 12.58; menadione (22.9%), 0.83; cyanocobalamin (1%), 0.94; D-biotin (2%), 0.75; folic acid (95%), 0.42; thiamine nitrate (98%), 0.11; ascorbic acetate (95%), 4.31; niacin (99%), 2.58; meso-inositol (98%), 19.39; calcium-D-pantothenate (98%), 2.56; riboflavin (80%), 0.63; pyridoxine hydrochloride (98%), 0.62. All ingredients were diluted with maize starch to 1 kg. ^2^ Per kilogram of mineral premix (g/kg): MnSO_4_·H_2_O (31.8% Mn), 1.8900; MgSO_4_·H_2_O (15.0% Mg), 200.0000; FeSO_4_·H_2_O (30.0% Fe), 24.5700; ZnSO_4_·H_2_O (34.5% Zn), 8.2500; CuSO_4_·5H_2_O (25.0% Cu), 0.9600; KI (76.9% I), 0.0668; Na_2_SeO_3_ (44.7% Se), 0.0168. All ingredients were diluted with maize starch to 1 kg. ^3^ Vitamin A premix: premix was added to obtain graded level of vitamin A and the amount of maize starch was reduced to compensate. ^4^ Crude protein and crude lipid contents were measured value. ^5^ Available phosphorus, n-3, and n-6 contents were calculated according to NRC [19].

**Table 2:** Real-time PCR primer sequences.

Genes	Forward (5′→3′)	Reverse (5′→3′)	Temperature (°C)	Accession Number
*CuZnSOD*	CGCACTTCAACCCTTACA	ACTTTCCTCATTGCCTCC	61.5	GU901214
*MnSOD*	ACGACCCAAGTCTCCCTA	ACCCTGTGGTTCTCCTCC	60.4	GU218534
*CAT*	GAAGTTCTACACCGATGAGG	CCAGAAATCCCAAACCAT	58.7	FJ560431
*GPx1a*	GGGCTGGTTATTCTGGGC	AGGCGATGTCATTCCTGTTC	61.5	EU828796
*GPx1b*	TTTTGTCCTTGAAGTATGTCCGTC	GGGTCGTTCATAAAGGGCATT	60.3	KT757315
*GPx4a*	TACGCTGAGAGAGGTTTACACAT	CTTTTCCATTGGGTTGTTCC	60.4	KU255598
*GPx4b*	CTGGAGAAATACAGGGGTTACG	CTCCTGCTTTCCGAACTGGT	60.3	KU255599
*GSTr*	TCTCAAGGAACCCGTCTG	CCAAGTATCCGTCCCACA	58.4	EU107283
*GSTp1*	ACAGTTGCCCAAGTTCCAG	CCTCACAGTCGTTTTTTCCA	59.3	KM112099
*GSTp2*	TGCCTTGAAGATTATGCTGG	GCTGGCTTTTATTTCACCCT	59.3	KP125490
*GR*	GTGTCCAACTTCTCCTGTG	ACTCTGGGGTCCAAAACG	59.4	JX854448
*Nrf2*	CTGGACGAGGAGACTGGA	ATCTGTGGTAGGTGGAAC	62.5	KF733814
*keap1a*	TTCCACGCCCTCCTCAA	TGTACCCTCCCGCTATG	63.0	KF811013
*keap1b*	TCTGCTGTATGCGGTGGGC	CTCCTCCATTCATCTTTCTCG	57.9	KJ729125
*TOR*	TCCCACTTTCCACCAACT	ACACCTCCACCTTCTCCA	61.4	JX854449
*S6K1*	TGGAGGAGGTAATGGACG	ACATAAAGCAGCCTGACG	54.0	EF373673
*β-actin*	GGCTGTGCTGTCCCTGTA	GGGCATAACCCTCGTAGAT	61.4	M25013

CuZnSOD = copper/zinc superoxide dismutase; MnSOD = manganese superoxide dismutase; CAT = catalase; GPx1a = glutathione peroxidase 1a; GPx1b = glutathione peroxidase 1b; GPx4a = glutathione peroxidase 4a; GPx4b = glutathione peroxidase 4b; GSTr = glutathione-S-transferase r; GSTp1 = glutathione-S-transferase p1; GSTp2 = glutathione-S-transferase p2; GR = glutathione reductase; Nrf2 = nuclear factor erythroid 2-related factor 2; Keap1a = Kelch-like ECH-associated protein 1a; Keap1b = Kelch-like ECH-associated protein 1b; TOR = target of rapamycin; S6K1 = ribosomal protein s6 kinase polypeptide 1.

**Table 3:** Effects of dietary vitamin A on muscle proximate composition and physicochemical characteristics of on-growing grass carp ^1^.

	Dietary VA Levels, IU/kg Diet						SEM	*p*-Values	
	18.69	606.8	1209	1798	2805	3796		Linear	Quadratic
Moisture, %	79.67 ^a^	78.16 ^b^	77.75 ^bc^	77.15 ^c^	77.93 ^bc^	78.04 ^bc^	0.20	0.0001	<0.0001
Protein, %	15.20 ^d^	16.51 ^c^	17.11 ^b^	17.78 ^a^	17.00 ^b^	17.02 ^b^	0.06	<0.0001	<0.0001
Lipids, %	10.06 ^b^	10.92 ^ab^	12.07 ^a^	12.17 ^a^	11.94 ^a^	11.15 ^ab^	0.31	0.01	<0.01
Cooking loss, %	16.03 ^a^	12.56 ^b^	10.12 ^c^	10.25 ^c^	12.25 ^b^	13.85 ^b^	0.39	<0.01	<0.0001
Shear force, N	1.18 ^d^	1.30 ^c^	1.53 ^a^	1.51 ^a^	1.42 ^b^	1.31 ^c^	0.02	<0.0001	<0.0001
pH_24h_	6.43 ^b^	6.54 ^b^	6.72 ^a^	6.79 ^a^	6.77 ^a^	6.53 ^b^	0.04	<0.01	<0.0001
Hydroxyproline, mg/g tissue	0.38 ^d^	0.46 ^b^	0.58 ^a^	0.57 ^a^	0.43 ^bc^	0.42 ^c^	0.01	0.09	<0.0001
Carnosine, ng/g tissue	348.51 ^c^	409.53 ^bc^	485.00 ^a^	485.22 ^a^	489.94 ^a^	431.10 ^ab^	14.96	0.0002	<0.0001
Lactic acid, mmol/g protein	2.58 ^a^	2.16 ^b^	2.09 ^b^	1.63 ^d^	1.86 ^c^	1.85 ^c^	0.04	<0.0001	<0.0001
Cathepsin B, U/g protein	3.98 ^a^	3.57 ^b^	2.90 ^c^	2.89 ^c^	3.23 ^bc^	3.29 ^b^	0.08	<0.0001	<0.0001
Cathepsin L, U/g protein	1.85 ^a^	1.72 ^b^	1.53 ^cd^	1.44 ^d^	1.54 ^cd^	1.58 ^c^	0.03	<0.0001	<0.0001

^1^ Data are means of three replicate groups, two fish for each replicate (*n* = 3), SEM = standard error of the mean. ^a,b,c,d^ within a row, means without a common lowercase superscript differ (*p* < 0.05).

**Table 4:** Effects of dietary vitamin A on muscle amino acid composition (mg/100 g dry) of on-growing grass carp ^1^.

	Dietary VA Levels, IU/kg Diet						SEM	*p*-Values	
	18.69	606.8	1209	1798	2805	3796		Linear	Quadratic
Glu	3.89 ^d^	4.13 ^c^	4.34 ^ab^	4.52 ^a^	4.20 ^bc^	4.20 ^bc^	0.04	0.0001	<0.0001
Asp	2.10	2.05	2.11	2.07	2.08	2.08	0.04	0.96	0.89
Gly	25.27	24.80	24.88	24.93	25.36	25.46	0.63	0.62	0.48
Ser	3.66	3.74	3.79	3.70	3.68	3.71	0.09	0.98	0.53
Ala	11.74	11.37	11.54	11.43	11.68	11.59	0.27	0.98	0.50
Met	5.13 ^b^	5.49 ^b^	5.82 ^ab^	6.26 ^a^	5.73 ^ab^	5.76 ^ab^	0.15	<0.01	<0.01
Thr	9.46 ^b^	10.84 ^a^	11.51 ^a^	11.76 ^a^	11.40 ^a^	11.42 ^a^	0.25	<0.01	<0.01
Lys	31.19 ^c^	34.91 ^bc^	38.82 ^ab^	40.19 ^a^	38.32 ^ab^	37.83 ^ab^	0.95	0.0001	0.0003
Arg	19.56 ^c^	21.15 ^bc^	23.76 ^a^	23.61 ^a^	22.13 ^ab^	22.11 ^ab^	0.49	<0.01	0.0002
His	169.71	173.39	177.13	174.86	173.25	172.57	2.62	0.61	0.10
Val	6.17	6.29	6.22	6.28	6.31	6.32	0.15	0.52	0.92
Ile	2.14	2.20	2.26	2.22	2.16	2.19	0.05	0.77	0.24
Leu	3.09	3.06	3.13	3.10	3.14	3.12	0.07	0.55	0.89
Phe	3.34	3.25	3.30	3.24	3.23	3.28	0.07	0.50	0.53
Tyr	5.21	5.21	5.24	5.23	5.24	5.20	0.11	0.95	0.80

^1^ Data are means of three replicate groups, two fish for each replicate (*n* = 3), SEM = standard error of the mean. ^a,b,c,d^ within a row, means without a common lowercase superscript differ (*p* < 0.05).

**Table 5:** Effect of dietary vitamin A on the fillet fatty acid (FA) profile (% of total FA methyl esters) of on-growing grass carp ^1^.

	Dietary VA Levels, IU/kg Diet						SEM	*p*-Values	
	18.69	606.8	1209	1798	2805	3796		Linear	Quadratic
C14: 0	4.56	4.56	4.64	4.54	4.74	4.91	0.12	0.05	0.25
C15: 0	0.26	0.26	0.27	0.26	0.26	0.27	0.01	0.60	0.49
C16: 0	23.00 ^ab^	23.22 ^a^	22.61 ^ab^	21.23 ^b^	21.22 ^b^	22.05 ^ab^	0.39	<0.01	0.15
C17: 0	0.24	0.25	0.24	0.24	0.25	0.25	0.02	0.85	0.85
C18: 0	5.59	5.63	5.42	5.07	5.39	5.61	0.13	0.36	<0.05
C20: 0	0.23	0.23	0.23	0.22	0.23	0.22	0.01	0.49	0.91
C23: 0	0.28	0.29	0.29	0.28	0.29	0.31	0.02	0.28	0.72
C24: 0	0.77	0.78	0.75	0.74	0.82	0.82	0.04	0.29	0.24
C14: 1	0.21	0.22	0.21	0.22	0.26	0.25	0.02	0.10	0.86
C16: 1	14.02 ^a^	12.94 ^ab^	12.27 ^bc^	11.69 ^bc^	11.70 ^bc^	11.55 ^c^	0.28	<0.0001	<0.05
C17: 1	0.38	0.35	0.37	0.34	0.33	0.38	0.02	0.73	0.11
C18: 1c + t	22.51	23.20	23.70	24.37	24.57	23.52	0.54	0.05	0.07
C20: 1	1.88	1.82	1.80	1.84	1.76	1.70	0.05	<0.05	0.59
C22: 1	0.20	0.19	0.20	0.21	0.21	0.19	0.01	0.98	0.32
C18: 2c + t	7.83	7.78	7.99	8.50	8.52	8.03	0.18	<0.05	0.09
C20: 2	0.43	0.43	0.40	0.40	0.41	0.38	0.02	0.06	0.97
C18: 3n − 6	0.67	0.65	0.67	0.66	0.66	0.64	0.02	0.53	0.63
C18: 3n − 3	4.60 ^b^	5.00 ^ab^	5.03 ^ab^	5.40 ^ab^	5.56 ^a^	5.54 ^a^	0.19	<0.01	0.37
C20: 3n − 6 + C21: 0	0.37	0.39	0.37	0.38	0.38	0.35	0.02	0.38	0.41
C20: 3n-3	1.06 ^b^	1.10 ^ab^	1.19 ^ab^	1.20 ^ab^	1.25 ^a^	1.21 ^ab^	0.04	<0.01	0.13
C20: 4	0.41	0.42	0.40	0.40	0.40	0.39	0.02	0.25	0.93
C20: 5 + C22: 0	1.11	1.08	1.04	1.03	1.06	1.00	0.04	0.13	0.79
C22: 6	9.39 ^b^	9.20 ^b^	9.92 ^ab^	10.78 ^a^	10.14 ^ab^	10.42 ^ab^	0.27	<0.01	0.24
ΣSFA	34.94 ^a^	35.22 ^a^	34.46 ^ab^	32.58 ^b^	33.19 ^ab^	34.45 ^ab^	0.48	<0.05	<0.05
ΣUFA	64.65 ^b^	64.36 ^b^	65.15 ^ab^	67.02 ^a^	66.41 ^ab^	65.17 ^ab^	0.48	<0.05	<0.05
ΣMUFA	39.19	38.73	38.53	38.66	38.83	37.59	0.49	0.09	0.59
ΣPUFA	25.87 ^b^	26.04 ^b^	27.01 ^ab^	28.76 ^a^	27.98 ^ab^	27.97 ^ab^	0.45	<0.01	0.08

^1^ Data are means of three replicate groups, two fish for each replicate (*n* = 3), SEM = standard error of the mean. ^a,b,c^ within a row, means without a common lowercase superscript differ (*p* < 0.05). ΣSFA = Total saturated fatty acid; ΣUFA = Total unsaturated fatty acid; ΣMUFA = Total monounsaturated fatty acid; ΣPUFA = Total polyunsaturated fatty acid.

**Table 6:** Effect of dietary vitamin A on antioxidant parameters in muscle of on-growing grass carp ^1^.

	Dietary VA Levels, IU/kg Diet						SEM	*p*-Values	
	18.69	606.8	1209	1798	2805	3796		Linear	Quadratic
ASA, U/g protein	75.76 ^c^	83.48 ^bc^	96.85 ^a^	92.20 ^ab^	82.36 ^bc^	82.68 ^bc^	2.33	0.20	<0.0001
AHR, U/mg protein	89.54 ^c^	94.78 ^abc^	99.75 ^ab^	101.54 ^a^	91.49 ^bc^	90.89 ^c^	1.83	0.93	0.0002
CuZnSOD, U/mg protein	2.83 ^c^	2.96 ^bc^	3.36 ^ab^	3.65 ^a^	3.46 ^a^	3.30 ^ab^	0.09	0.0001	0.0005
MnSOD, U/mg protein	3.82 ^b^	4.30 ^ab^	4.35 ^ab^	4.48 ^a^	4.20 ^ab^	4.17 ^ab^	0.12	0.14	<0.01
CAT, U/mg protein	1.06 ^c^	1.28 ^b^	1.48 ^a^	1.64 ^a^	1.61 ^a^	1.62 ^a^	0.04	<0.0001	<0.0001
GPx, U/mg protein	84.96 ^b^	93.14 ^ab^	103.73 ^a^	104.63 ^a^	104.43 ^a^	103.25 ^a^	2.92	0.0002	<0.01
GST, U/mg protein	49.17	50.11	51.64	51.14	50.11	50.19	1.38	0.70	0.27
GR, U/g protein	17.44 ^b^	19.64 ^ab^	19.70 ^ab^	22.06 ^a^	21.35 ^ab^	20.54 ^ab^	0.83	<0.01	<0.05
GSH, mg/g protein	1.33 ^b^	1.50 ^ab^	1.63 ^a^	1.62 ^a^	1.62 ^a^	1.60 ^a^	0.04	0.0003	<0.01
Vitamin A, μg/kg tissue	1.63 ^c^	5.43 ^b^	6.74 ^ab^	5.84 ^ab^	6.56 ^ab^	8.29 ^a^	0.52	<0.0001	<0.05

^1^ Data are means of three replicate groups, two fish for each replicate (*n* = 3), SEM = standard error of the mean. ^a,b,c^ within a row, means without a common lowercase superscript differ (*p* < 0.05). AHR = anti-hydroxyl radical; ASA = anti-superoxide anion; CuZnSOD = copper/zinc superoxide dismutase; MnSOD = manganese superoxide dismutase; CAT = catalase; GPx = glutathione peroxidase; GST = glutathione-S-transferase; GR = glutathione reductase; GSH = glutathione.

**Table 7:** Correlations of different indices in the muscle of on-growing grass carp.

Dependent Parameters	Independent Parameters	r	*p*
pH_24h_	Lactic acid content	−0.758	=0.08
Shear force	Hydroxyproline content	+0.894	<0.05
	Cathepsin B activity	−0.964	<0.01
	Cathepsin L activity	−0.906	<0.05
MnSOD activity	*MnSOD* mRNA level	+0.928	<0.01
CAT activity	*CAT* mRNA level	+0.980	<0.01
GPx activity	*GPx4a* mRNA level	+0.999	<0.01
	*GPx4b* mRNA level	+0.936	<0.01
*MnSOD* mRNA	*Nrf2* mRNA level	+0.938	<0.01
*GPx1a* mRNA		+0.842	<0.05

MnSOD = manganese superoxide dismutase; CAT = catalase; GPx = glutathione peroxidase; Nrf2 = nuclear factor erythroid 2-related factor 2.

## Data Availability

Data are contained within the article.
